# Factors contributing to rapidly increasing rates of cesarean section in Armenia: a partially mixed concurrent quantitative-qualitative equal status study

**DOI:** 10.1186/s12884-018-2158-6

**Published:** 2019-01-03

**Authors:** Meri Tadevosyan, Anna Ghazaryan, Arusyak Harutyunyan, Varduhi Petrosyan, Adam Atherly, Kim Hekimian

**Affiliations:** 1 Gerald and Patricia Turpanjian School of Public Health, American University of Armenia, 40 Marshal Baghramian Ave, 0019 Yerevan, Armenia; 2 Health Services Research Center, Larner College of Medicine, University of Vermont, 89 Beaumont Ave, 89 Beaumont Ave, Burlington, VT 05405 USA; 3 Department of Pediatrics, Institute of Human Nutrition, Columbia University, 630 West 168th St., PH 1512 East, New York City, NY 10032 USA

**Keywords:** Cesarean section, Vaginal birth, Financial reimbursement

## Abstract

**Background:**

Armenia has an upward trend in cesarean sections (CS); the CS rate increased from 7.2% in 2000 to 31.0% in 2017. The purpose of this study was to investigate potential factors contributing to the rapidly increasing rates of CS in Armenia and identify the actual costs of CS and vaginal birth (VB), which are different from the reimbursement rates by the Obstetric Care State Certificate Program of the Ministry of Health.

**Methods:**

This was a partially mixed concurrent quantitative-qualitative equal status study. The research team collected qualitative data via in-depth interviews (IDI) with obstetrician-gynecologists (OBGYN) and policymakers and focus group discussions (FGD) with women. The quantitative phase of the study utilized the bottom-up cost accounting (considering only direct variable costs) from the perspective of providers, and it included self-administered provider surveys and retrospective review of mother and child hospital records. The survey questionnaire was developed based on IDIs with providers of different medical services.

**Results:**

The mean estimated direct variable cost per case was 35,219 AMD (94.72 USD) for VB and 80,385 AMD (216.19 USD) for CS. The ratio of mean direct variable costs for CS vs. VB was 2.28, which is higher than the government’s reimbursement ratio of 1.64. The amount of bonus payments to OBGYNs was 11 fold higher for CS than for VB indicating that OBGYNs may have significant financial motivation to perform CS without a medical necessity. The qualitative study analysis revealed that financial incentives, maternal request and lack of regulations could be contributing to increasing the CS rates. While OBGYNs did not report that higher reimbursement for CS could lead to increasing CS rates, the policymakers suggested a relationship between the high CS rate and the reimbursement mechanism. The quantitative phase of the study confirmed the policymakers’ concern.

**Conclusion:**

The study suggested an important relationship between the increasing CS rates and the current health care reimbursement system.

## Background

Cesarean section (CS) can be an important, lifesaving procedure for both the mother and the baby in certain medical conditions [1]. However, unnecessary CS can lead to increased medical risks for both mothers and infants. The World Health Organization recommends a CS rate of 15% or less to balance the benefits and risks of CS [2]. In addition to the health consequences of high rates of CS, it also puts an additional financial burden on health care systems, particularly in low- and middle-income countries (LMIC) [3].

The incidence rates of CS varies widely worldwide [4, 5]. Many countries are taking measures to reduce and/or prevent the increase of CS rates to meet the World Health Organization recommendation [2, 6, 7]. However, the CS rates in some countries are significantly above the WHO recommendation, e.g., Turkey (50% of births), Mexico (45%), Chile (45%), Italy (36%), and the USA (32%). In contrast, other countries, including Iceland (15%), Israel (15%), Sweden (16%) and Norway (17%), have CS rates at or near the recommendation [6].

CS is a major surgical procedure and carries health risks for both mothers and infants. Compared with vaginal birth (VB), CS without medical indication is associated with greater chance of maternal mortality, infection, hemorrhage, adhesions, bleeding and lacerations, bleeding in a subsequent pregnancy, extended hospital stay and/or recovery time, reactions to medication, risk of additional surgeries and negative emotional reactions for mothers [8–10]. In addition, infants delivered by CS are at higher risk of having breathing problems, respiratory distress, low APGAR score, fetal injury, allergic rhinitis, food allergy, childhood asthma and childhood onset of type1 diabetes compared with those delivered by VB [11–14].

Patient and obstetrician-gynecologist (OBGYN) related factors could contribute to elevated rates of CS. Previous research had identified a number of factors that lead to high CS rates, including policies promoting subsequent CS and discouraging vaginal birth after cesarean, technological monitoring of labor, fear of malpractice suits in case of breech or forceps deliveries, childbearing patterns (older age of mothers), and reimbursement mechanisms [15]. The final decision maker whether to perform a CS versus VB is the OBGYN [16].

The financial motivation - higher reimbursement for CS than for VB – has been identified as one the main determinants for increasing CS rates [2]. The ratio of expenses for CS versus VB differs across countries: it is very high (2.8–5.0 times) in LMIC (including Pakistan and some countries in Africa and Latin America) and much lower (1.1–1.8 times) in high-income countries (including Australia, Portugal, Israel, Canada, the USA and England) [17–21]. Reimbursement mechanisms that pay more for CS than VB can incentivize hospitals to perform more CS [22]. Some insurance companies in the USA reimburse only for “medically necessary” services, and OBGYNs justify CS by mentioning medical indications (even if they are absence) in the medical records to be reimbursed by insurance companies [23]. Similarly, in Australia, despite existing regulations against CS without medical indications, most doctors in many public hospitals, perform such deliveries [24].

Historically, MOH funding for maternity services in Armenia was below the real cost. In 2006, 91% of women who gave birth made informal payments to the medical staff, including OBGYNs and nurses [25]. In response to this problem, the Ministry of Health (MOH) of Armenia launched the Obstetric Care State Certificate Program in July 2008, a national policy which pays for all obstetric services, including CS, for all pregnant women in the country [26]. The new policy offers certificates (vouchers) to all pregnant women when they reach the 22nd week of pregnancy. The certificates are given to the hospital of the woman’s choice, which then submits them to the MOH for reimbursement [26]. The MOH set the reimbursement rates for obstetric services based on input from providers and the heads of maternity hospitals, who provided estimated reimbursement levels that would eliminate informal payments [27]. The payment to hospitals per birth depends on the level of specialization of maternity hospitals, the type of delivery (CS vs. VB), and geographic location [28]. The ministerial order regulating the Obstetric Care State Certificate Program specifies that in addition to the base monthly remuneration the health providers should receive bonus payments for each birth [28]. The bonus payment was fixed for CS (25000AMD paid to OBGYN per CS) and left unregulated for VB [28].

Similar to what have been observed in high income countries [17, 18, 21, 28], the reimbursement for CS is about 1.7 times higher than for VB in Armenia. After the implementation of the policy (in 2008 to 2010), a sharp increase in CSs (from 18 to 24%) was observed in secondary level maternity hospitals of Yerevan, the capital city of Armenia; the change was smaller in tertiary level maternities (from 25 to 28%) [29, 30].

**Fig. 1:**
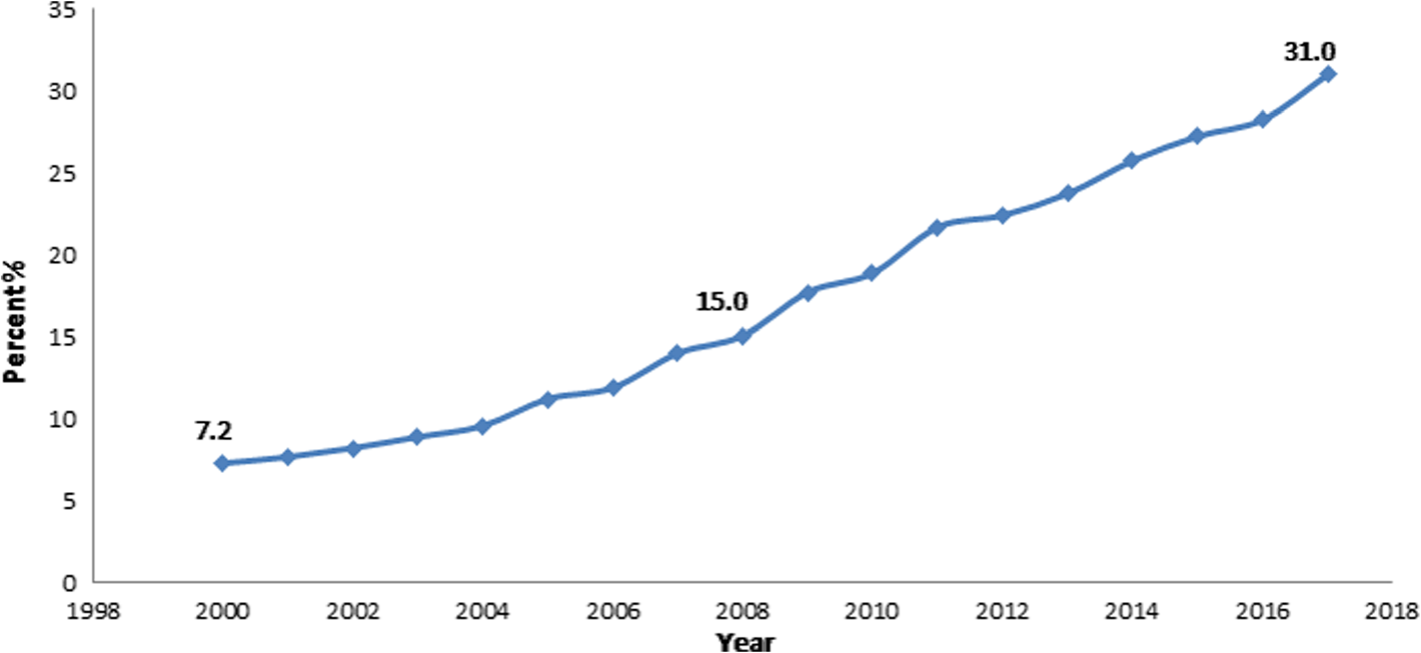
Cesarean section rates in Armenia (2000–2017)

It is not clear if the recent trends in Armenia are due to the Obstetric Care State Certificate Program or a reflection of a historical increasing trend. Armenia has experienced significant increases in CS rates over the past 17 years, from 7.2% in 2000 to 31.0% in 2017 (Fig. 1) [30]. Some OBGYNs suggested that the increase could be explained by a positive change in attitude toward CS among women who might consider it as an option to give birth without labor pain [27]. The CS rate is higher than the World Health Organization recommendation, suggesting that some of the CS are not medically necessary and might be influenced by the significantly higher reimbursement for CS vs. VB [27].

The purpose of this study was to investigate factors contributing to the rapidly increasing rates of CS in Armenia. We also wanted to explore if the actual costs for CS and VB are different from the reimbursement rates by the Obstetric Care State Certificate Program of the Ministry of Health. The results of the current study contribute to improving the regulatory mechanisms of CS and financing mechanisms of the Obstetric Care State Certificate Program.

## Methods

### Study design

The study utilized partially mixed concurrent quantitative-qualitative equal status study design. In this study design, quantitative and qualitative phases are implemented concurrently and have approximately equal weight [31, 32]. We conducted hospital-based cost accounting analysis and qualitative research with key informants simultaneously. The two components of the study were merged after data collection and analysis.

#### Quantitative cost accounting component

For the cost accounting study, the research team approached five of the six maternity hospitals located in Yerevan where the increase in CS rates was the highest [29, 33]; two of them agreed to participate. We obtained permission from the maternity hospital directors to conduct the study in their facility.

We applied a bottom-up cost accounting method, using the providers’ perspective on cost [34]. This method adds all the collected information about each cost input to obtain the total cost of treatment [34]. The research team considered direct variable costs including the costs of labor, medication, lab tests, and supplies.

To measure the cost, a survey instrument was developed based on ten in-depth interviews with different health providers. Information was collected about health providers’ contact time per case, performance-based bonus payment per case, and medical supplies/disposables used per case through a self-administered provider survey. Retrospective review of mother and child hospital records of those who gave birth in December 2010 added data about the mode of delivery, length of stay, medication and lab tests utilized for mother and childcare.

The estimate sample size for the record review was 70, considering the proportions of CS and VB in secondary level maternities, Z = 1.96 for 95% confidence interval, and the desired margin of error d = 0.1. We selected 35 medical records from each maternity hospital (8 CS (24%) and 27 VB (76%)) through a frequency matched random sampling method [35]. From the total list of medical records for the specified time period, the team randomly selected eight CS records from each hospital and matched each of them with the next three or four VB records (*n* = 27 per hospital). Fifty-five providers of neonatal, surgical, delivery, and postpartum services from two hospitals (*n* = 29 and *n* = 26) agreed to participate and answer the self-administered questionnaire.

#### Qualitative component

The qualitative component of the study targeted 1) women who underwent CS at least three months before the study, 2) women who had vaginal delivery at least three months before the study, 3) first time pregnant women at the time of the study, 4) OBGYNs from maternity hospitals, 5) OBGYNs working in polyclinics (polyclinics are primary care facilities that provide outpatient services) [36], and 6) policymakers [37]. All the participants were from the capital city Yerevan. We collected data through three focus group discussions (FGD) with women and IDIs with OBGYNs and policymakers. The main topics discussed during the FGDs referred to personal birth experience (preferences in the mode of delivery and factors that influence their choice of delivery mode, satisfaction, and whether they made any out of pocket payments), knowledge about advantages and disadvantages of CS and VB, attitudes toward CS on maternal request.

The main topics discussed during the IDIs with OBGYNs referred to the attitudes toward the increasing CS rate in Armenia, potential factors influencing increasing CS rate, influence of new payment system on the increasing CS rate and attitudes toward CS on maternal request and common practice of performing CS on maternal request, main reasons for having CS on maternal request, suggestions to decrease CS rate, discussion of delivery methods during antenatal visits with women, and factors that could influence women’s decision.

The main topics discussed during the IDIs with policymakers referred to the attitudes toward the increasing CS rate in Armenia, impact of the Obstetric Care State Certificate Program on the increased CS rate, attitudes toward CS on maternal request, and existing mechanisms and suggestions to decrease financial losses due to CS without medical indications.

Women with poor knowledge of Armenian and delivery of the newborn with health problems were excluded from FGDs. We used convenience sampling methodology for participants’ recruitment.

The research team developed seven different guides for IDIs and FGDs with different participant groups for the qualitative study. The guides were pre-tested and revised to improve them. We also developed a demographic information form for the participants. After obtaining the participants’ approval we audio-recorded the interviews for transcribing purposes. Three people refused to be recorded and the research team only took notes during the interviews.

### Data management and analysis

#### Quantitative cost accounting component

We classified the resource items into the following groups: medical supplies/disposables, medication, and lab tests. Then we placed monetary value on the listed resources to estimate the mean direct variable costs of CS and VB, using the wholesale current prices (recently updated, lowest price) of medicines and medical supplies/disposables from Pharm-Info, which is an automated electronic system of sales for drugstores [38]; for laboratory tests, we used the unit cost of each performed laboratory test from the hospital price lists.

Based on providers’ reports, the research team estimated the mean performance-based bonus compensation per case and the mean contact time per case for each type of health professional providing VB and CS. We estimated the labor cost per case for VB and for CS considering the mean contact time per case and using the highest base monthly remuneration scale for each type of health provider (including midwifes and nurses) involved in providing VB and CS suggested by the Ministry of Health [28]. Typical basic monthly salary for OBGYNs, without additional payments for night shifts, vacations, overtime and holidays, ranges from 25,000 AMD (67.2 USD) to 60,000 AMD (161.3 USD) [28]. According to the Labor Code of the Republic of Armenia, the normal workload cannot exceed 40 h per week. The maximum duration of working time, including overtime work, cannot exceed 12 h per day (including breaks), and 48 h per week. However, the duration of working time of specific categories of workers, such as healthcare providers working on an uninterrupted shift basis, may be 24 h a day. The average duration of the providers’ working hours cannot exceed 48 h per week, and the rest time between the working days cannot be less than 24 h [39].

The research team estimated the average labor cost per hour dividing the monthly remuneration for each health provider by the average number of working days in one month (21.667) and by eight working hours per day. To calculate the labor cost per case, we multiplied the cost per hour by contact time per case. We did not calculate the labor cost for anesthesiologist, anesthesiologist nurse, operation room nurse and intensive care nurse, because they received only a bonus payment per case. The study identified the ratios of direct variable costs of CS and VB to compare them with the actual ratio of reimbursement.

#### Qualitative component

The research team analyzed the IDI and FGD transcripts using the directed content (deductive) analysis approach [40]. The main themes in the directed content analysis were predefined based on the research questions and literature review. The complete data was read and relevant paragraphs were highlighted. The next step was the coding of highlighted parts of the transcripts according to predetermined codes. If any data could not be categorized within the initial coding scheme, a new code was assigned. The researcher who performed the qualitative data collection also conducted the coding and the initial analysis. The other researchers, who were not involved in data collection, reviewed the coding, provided feedback and contributed to the analysis. The study findings were analyzed with the following themes: 1) financial incentives for CS and 2) maternal request for CS without medical indications.

### Ethical aspects

The AUA Institutional Review Board #1 reviewed and approved the study protocols and verbal consent forms. Each study participant gave a verbal consent before participation.

## Results

### Results of the secondary level hospital-based cost accounting study

The mean estimated direct variable cost per case was 80,385 AMD (216.19 USD) for CS and 35,219 AMD (94.72 USD) for VB or the mean cost ratio of 2.28 (Tables 1 and 2). The direct variable cost per case included the cost of medications, laboratory tests, supplies/disposables and labor.

**Table 1:** Mean cost of medicines, tests, and medical supplies/disposables utilized per case, mean cost ratios (C*V*/VB) and mean length of hospital stay

Vaginal Birth			C-Section		
Resource items	Mean cost (VB) AMD (USD*)	Range (between two hospitals) AMD (USD)	Mean cost (CS) AMD (USD)	Range (between two hospitals) AMD (USD)	Ratios of mean costs (CV/VB)^a^
Medicines	1559 (4.19)	465 (1.25) - 2653 (7.13)	6903 (18.56)	6483 (17.43) - 7323 (19.69)	4.42
Tests	9524 (25.61)	9030 (24.28) - 10,020 (26.94)	20,037(53.88)	18,425 (49.55) - 21,650 (58.22)	2.10
Medical supplies/disposables	15,654 (42.10)	12,875 (34.62)-18,430 (49.56)	43,840 (117.90)	39,550 (106.36) - 48,130 (129.44)	2.80
Mean length of hospital stay (day)	4.2		6.4		1.52

^*^$1 = AMD 371.82 (as of 03/05/2011, date of getting information on the wholesale current prices of medicines and medical supplies/disposables from the Pharm-Info automated electronic system of sales for drugstores)

^a^The government’s reimbursement ratio is 1.64

**Table 2:** Mean contact time, mean labor cost, mean bonus payment per case for each specialist by mode of delivery and ratios of labor cost (CV/VB)

Specialist	Mean Time	Mean cost AMD (USD)	Mean bonus AMD (USD)	Ratios of labor cost
Vaginal Birth				
Obstetrician-gynecologist	410 min	2365 (6.36)	2088 (5.61)	0.71
Delivery room midwife	480 min	1938 (5.21)	659 (1.77)	
Anesthesiologist nurse	30 min		350 (0.94)	
Anesthesiologist	30 min		1300 (3.49)	
Postpartum care midwife	426 min	1638 (4.40)	239 (0.64)	2.11
Neonatal care nurse	432 min	1744 (4.69)	239 (0.64)	1.39
Neonatologist	138 min	796 (2.14)	692 (1.86)	1.48
Total	1956 min	8482 (22.81)	5566 (14.96)	
C-Section				
Obstetrician-gynecologist	290 min	1673 (4.50)	23,875 (64.21)	0.71
Anesthesiologist nurse	150 min		2250 (6.05)	
Anesthesiologist	186 min		8000 (21.51)	
Postpartum care midwife	900 min	3461 (9.30)	393 (1.05)	2.11
Neonatal care nurse	600 min	2423 (6.51)	314 (0.84)	1.39
Neonatologist	204 min	1182 (3.17)	2667 (7.17)	1.48
Operation room nurse	90 min		2250 (6.05)	
Intensive care nurse	1620 min		1500 (4.03)	
Assistant	150 min	865 (2.32)	4285 (11.52)	
Total	4200 min	9605 (25.83)	45,533 (117.08)	

The costs associated with medicines, tests, and supplies were consistently higher for CS than for VB: the mean cost ratios were 4.4 for medicines, 2.1 for tests and 2.8 for medical supplies and disposables (Table 1). The mean length of stay for CS was 6.4 days, compared to4.2 days for VB - the mean length of stay ratio of 1.52 (Table 1). The ratio of contact time and labor costs per case for health providers that are involved in either CS or VB ranged from 0.71 for OBGYNs to 2.11 for postpartum care midwives (Table 2).

The ratio of mean bonus payments for each birth to an OBGYN was 11.44 for providing CS vs. VB, ranging from 6.90 to 42.70 between hospitals. It was 1.64 to a postpartum midwife, 1.31 to a neonatal care nurse, 3.86 to a neonatologist, 6.15 to an anesthesiologist and 6.42 to an anesthesiologist-nurse (Table 3).

**Table 3:** Mean bonus ratios (CV/VB)

Performance based compensation per case	Ratios of mean bonus	Range (between two hospitals)	Reimbursement ratio
Obstetrician-gynecologist	11.44	6.90–42.70	1.64
Neonatologist	3.86	3.00–6.10	1.64
Neonatal care nurse	1.31	1.30–1.30	1.64
Postpartum care midwife	1.64	1.10–1.80	1.64
Anesthesiologist	6.15	6.15–6.15	1.64
Anesthesiologist nurse	6.42	0.00–3.57	1.64

### Results of the qualitative study

The three FGDs included nine women with previous vaginal delivery, 10 with previous cesarean delivery, and eight women expecting their first child (Table 4). The mean duration of FGDs was 65 min. The mean age of women participants of the qualitative study was 28 (ranging from 21 to 38). Most of them had university education, four had vocational education and two had high-school education. Seventeen of them worked but were on maternity leave at the time of discussion. Their number of children ranged from 0 to three, and the mean age of children was nine months (three months to 18 months). Table 4 presents the demographic characteristics of FGD participants.

**Table 4:** Demographic characteristics of women, who participated in focus group discussions

Women	N of women	Mean age	Married	University ed.	Vocational ed.	High school	Employed	Mean N of kids	Mean age of kids in months	Mean discussion time
First time pregnant	8	26	8	6	1	1	5	0	N/A	64
Had VB	9	28	9	7	0	2	4	2	10	66
Had CS	10	30	10	8	1	1	8	1.5	8	67
Total	27	28	27	21	2	4	17	1.75	10	65

We also conducted 10 IDIs with OBGYNs and two with policymakers. The mean duration of IDIs was 20 min. All the OBGYNs involved in the qualitative study were women, five OBGYNs worked in polyclinics and the other five worked in maternity hospitals. Their mean age was 41 and the mean work experience was 12 years. To protect policymakers’ identity, we did not describe their demographic characteristics.

#### Financial incentives for CS

Almost all doctors participating in the study stated that they did not believe there was a relationship between the new payment system and increasing CS rates. The OBGYNs interviewed reported that the reimbursements for CS delivery did not outweigh the risks carried by the surgery, and did not feel that the monetary difference was a motivation for performing CS.

One OBGYN working in a hospital stated, ***“Stress related to performing the surgery is so high that I think a doctor who has some self-respect will not perform this surgery for 30,000 AMD*** **[approximately 80USD]...**^1^ ***I do not think that it [the CS rate] is related to*** **[higher reimbursements for CS].”**

The OBGYNs mainly explained the increasing CS rate by the increasing number of absolute and relative medical indications for CS in the recent years (e.g., breech presentation or assisted conception). Only one OBGYN working in a polyclinic suggested that OBGYNs working in hospitals could have a financial motivation to perform CS, ***“That might be the case*** **[having financial motivation]** ***for doctors working in hospitals. We*** **[polyclinic OBGYNs]** ***are not motivated in advising to go for a cesarean section …***”.

Contrary to the OBGYNs interviewed, the policymakers suggested that financial motivation could be a reason behind the increasing CSs. The policymakers stated that some OBGYNs (performing both VBs and CSs) earned up to 1,800,000 AMD per month (approximately 4864USD), which can be a reason for performing unnecessary CS.

One of the policymakers suggested that OBGYNs have a motivation to perform more CSs to receive more money, otherwise their income would be low due to lower number of births in their facilities, ***“We monitor the cesarean section rates and we know that there is no increase in cesarean section rates in the tertiary level facilities where the most complicated cases are referred to. On the other hand, we have observed increasing cesarean section rates in the secondary level facilities which, because of fewer number of births, could have a motivation to get more money.”***

The policymakers expressed an opinion that OBGYNs might also be interested in performing CSs since patients might feel obliged to make an out-of-pocket payment to show gratitude to an OBGYN in case of a surgery rather than a VB. One policymaker said, ***“For*** **cesarean section*, where out-of-pocket payments exist and are higher than for natural delivery, patients may feel obligated to pay a doctor a little “extra” for the surgery***.”

#### Maternal request for CS

OBGYNs reported an increase in pregnant women requesting CS without medical indication and, although they said they explained the disadvantages of having elective CS, they often took women’s preferences into consideration and performed a requested CS. For example, one maternity hospital-based OBGYN said, ***“If a woman wants a cesarean section she starts to cry too much and it leads to developing complications and affects the fetal heartbeat, which suffers, and finally she gets a cesarean section.”*** Another hospital-based OBGYN said, ***“In our hospital if women insist on a cesarean section we do it. It does not happen very often, but we do that …***”.

Most women in the FGDs said they favored VB, however, they also expressed the opinion that CS should be performed if a woman requests it. Participating women gave examples of cases when VB led to adverse outcomes (fetal hypoxia in labor or postpartum psychological issues among mothers) after OBGYNs refused to do a CS at the request of women, saying there were no medical indications for surgery. One first-time pregnant woman said, “***… if a woman insists on having a cesarean section despite all the efforts to change her mind, it must be performed. Otherwise, both the mother and child can have more harm than benefit from natural delivery.”*** A woman who delivered via CS confirmed this by saying, ***“I had a normal delivery with my first child. It was a huge stress for me, so I decided to have cesarean section during my next pregnancies. My doctor disagreed, as doctors believe that the second delivery is much easier … I insisted on having the cesarean section and I do not regret it.”***

An OBGYN working in a polyclinic refuted the idea that women could request for an elective CS. An OBGYN working in a polyclinic said, ***“Cesarean section is not performed without medical indications … There is a special part in the medical record named “indications” which must be filled in by the doctor …***”.

#### CS without medical indications

Most OBGYNs did not accept that they would perform CS without a medical indication explaining that the Ministry of Health strictly monitored the CS rates in each facility.

One of the policymakers confirmed this by saying, ***“We only reimburse cesarean section with medical indications. I have never seen in medical records an indication like ‘at woman’s request. If this was the case, we would not pay them money.”***

Some of the participants mentioned that they would need the chief doctor’s permission to perform such a CS. One OBGYN working in a polyclinic mentioned that OBGYNs would fabricate medical records to justify the CS, “***… they*** **[hospital doctors]** ***are writing something like hypoxia or placental abruption to justify the cesarean section*** **[performed without medical indications].*”***However, the OBGYNs highlighted that the existing guidelines do not explicitly specify the criteria according to which medically necessary CS could be performed, creating a loophole that OBGYNs use to justify CS without violating the existing guidelines. The policymakers stated that the last version of the guideline was written in 2000. One policymaker said, ***“We can compare our list of relative*** **[medical]** ***indications to the list of other countries or to World Health Organization recommendations*** **[for performing CS].** ***I do not think that there are huge differences. It was developed in 2000; before that time doctors used Soviet books or guides.”***

## Discussion

The study investigated the potential contributing factors to increasing cesarean section rates providing valuable information to policymakers to improve regulatory mechanisms of CS without medical indications and to improve the financing mechanism of the Obstetric Care State Certificate Program in Armenia. The qualitative study analysis revealed that three factors could be contributing to increasing CS rates: 1) financial incentives (reimbursement and out of pocket payments); 2) maternal request; and 3) lack of clear regulations. While OBGYNs did not express the opinion that higher reimbursements for CS could be a factor in increasing CS rates, the policymakers shared a concern about a potential link between the remuneration mechanism and increasing CS rate. The quantitative phase of the study confirmed the policymakers’ concern. The amount of bonus payments to OBGYNs was 11 fold higher for CS than for VB while the labor cost ratio was 0.71, indicating that OBGYNs, the main decision makers regarding the mode of delivery, have a very strong financial motivation to perform CS where it was not medically necessary. The MOH fixed the bonus amount for each CS and but not for each VB; as a result, in hospitals with a low total number of births, OBGYNs would receive a substantially lower bonus payment for VB and have a much stronger financial motivation to perform CS. This is consistent with research showing that secondary level facilities with lower numbers of births do indeed have higher CS rates [29]. This is also consistent with Liu et al. recent work demonstrating that the rate of CS performed by maternal request in secondary level facilities was higher than that of tertiary hospitals in China [41].

Another factor that could be linked to the financial motivation for increases in the CS rate was that patients who undergo CS are more likely to make an out-of-pocket payment to the OBGYN than patients with a VB because patients feel an obligation to make a thank you payment for the CS [25, 27]. According to the existing regulation, the MOH should reimburse only for medically indicated procedures [42], but some OBGYNs, policymakers and one woman interviewed reported that, in some hospitals doctors performed CS without medical indications and suggested that doctors likely fabricated the medical records to medically justify the performance of a CS. These findings are consistent with the study by Robson et al. who indicated that OBGYNs “disguise” indications for the CS in cases where it is performed by maternal request [24]. Most women participants favored VB and agreed that CS should be performed only in case of medical indications. However, many OBGYNs reported that some women requested to have a CS to avoid labor pain, postpartum lacerations and dilations, or to protect coital function. Reportedly, women with a history of difficult VB would particularly insist on having a CS. The international literature indicates that OBGYNs exaggerate women’s desire for CS to justify their performance of this more expensive procedure [43].

There are a number of possible reasons for the significant variation in care delivered by the two hospitals in our study. Variations of this type often signal quality of care issues [44]: in this case the differences may reflect the lack of standard treatment guidelines for the examined services.

The overall reimbursement ratio for CS, as compared to VB is 1.6 in Armenia. This ratio is more in line with higher income countries (1.4–1.8) than other LMIC [17, 18, 20, 21]. However, the estimated average cost ratio is higher than the reimbursement ratio. Improved resource management (including quality of obstetric and neonatal care services) could further reduce the cost ratio in Armenia. Given the cost ratio, an appropriate ratio between bonus payments would reduce the financial incentive to perform CS. The bonus payment ratio would ideally be comparable to the overall reimbursement ratio of 1.64.

Armenia also needs to adopt the international guidelines for practicing vaginal birth after CS to provide women with uterine scar an opportunity to have a labor trial in the next pregnancy. The Ministry of Health should develop alternative financing and regulatory mechanisms for performing CS without medical indications and based on only women’s request. The new regulatory mechanisms should clearly define CS on maternal request and identify a different payment mechanism for it (e.g., a formal co-payment covering the additional expenses for CS) allowing OBGYNs perform CS at “maternal request” and indicate it in medical records. This approach will decrease the unnecessary financial losses due to performing medically unnecessary CS. At the same time the co-payment for CS without medical indications could serve as a demand side mechanism to control the rates of CS. Along with this effort, the quality control mechanisms for obstetric and neonatal care services need to be improved prioritizing adherence to standard treatment guidelines for CS and VB; the treatment guidelines need to clearly specify the medical indications for CS. This mixed-method study provided the opportunity for triangulations between the qualitative and quantitative components, different data sources and participant groups. The strength of the quantitative component was the bottom-up costing approach. This method is considered more accurate measurement of resources used while providing medical service than the top-down approach [34].

Future cost analysis studies should increase the number and the scope of participating hospitals to include tertiary level maternities and maternities in marzes. Considering indirect fixed costs could also improve the accuracy of cost analyses.

This study did have a number of limitations. The quantitative component considered only direct variable costs. In addition, the cost accounting was conducted in only two secondary level maternity hospitals located in Yerevan limiting the generalizability of the study. Data collected through self-administered questionnaires could have recall bias. In addition, the medical records had different formats between hospitals. Although every effort was made to code consistently and only use data elements available in both record, there could be underlying data differences limiting the accuracy of data collected from them. Finally, the coding of the qualitative data was conducted by one researcher, but other members of the team checked it.

## Conclusions

This study demonstrates that facility and provider compensation can be an important contributing factor pushing CS rates upward and should be carefully considered while developing appropriate policies in both low-, middle-, and high-income countries. Health systems need to have specific financial and regulatory mechanisms for performing CS without medical indications to avoid forging medical records. Adherence to up-to-date standard treatment guidelines for VB and CS is important for assuring quality obstetric care.

## Abbreviations

AMD: Armenian Drams
CS: Cesarean Section
FGD: Focus Group Discussion
IDI: In-Depth Interview
LMIC: Low- and Middle-Income Countries
OBGYN: Obstetrician-Gynecologist
USA: United States of America
USD: United States Dollar
VB: Vaginal Birth
АUA: American University of Armenia

## Footnotes

1 According to the National Statistical Service of the Republic of Armenia the average monthly nominal salary in Armenia was AMD 102652 (USD 276) in 2010 and (January–April) 166,150 AMD (USD 343) in 2018.
